# Gastroesophageal Reflux Disease in the Young Population and Its Correlation With Anxiety and Depression

**DOI:** 10.7759/cureus.15289

**Published:** 2021-05-28

**Authors:** Pooja Bai, Shehar Bano, Sameet Kumar, Priyanka Sachdev, Ahmed Ali, Pariya Dembra, Parkash Bachani, Simra Shahid, Amna Jamil, Amber Rizwan

**Affiliations:** 1 Internal Medicine, Liaquat University of Medical and Health Sciences, Jamshoro, PAK; 2 Internal Medicine, University of Health Sciences, Lahore, PAK; 3 Internal Medicine, Chandka Medical College, Larkana, PAK; 4 Infectious Diseases, University of Louisville, Louisville, USA; 5 Internal Medicine, Jinnah Sindh Medical University, Karachi, PAK; 6 Obstetrics and Gynecology, Jinnah Postgraduate Medical Centre, Karachi, PAK; 7 Family Medicine, Jinnah Postgraduate Medical Centre, Karachi, PAK

**Keywords:** anxiety, depression, gerd, association, pakistan

## Abstract

Introduction

Stress and anxiety may disrupt normal GI function and lead to several GI disorders, including gastroesophageal reflux disease (GERD). In this study, we aimed to predict the prevalence of GERD in young patients and its association with anxiety and depression.

Material and Methods

This cross-sectional study enrolled 2,500 participants from the general public, with an age range of 18 to 40 years. Diagnosis of GERD was made via the Frequency Scale for the Symptoms of GERD (FSSG) questionnaire. The Hospital Anxiety and Depression Scale (HADS) was used to assess anxiety and depression.

Results

GERD was diagnosis in 401 (16.0%) participants. Anxiety was significantly more common in participants with GERD compared to participants without GERD (40.3% vs. 19.5%; p < 0.01). Similarly, participants with GERD had a higher prevalence of depression compared to participants without GERD (42.6% vs. 18.3%; p < 0.01).

Conclusion

GERD is highly prevalent among the young population. Anxiety and depression are significantly more prevalent in patients with GERD. Hence, the young population must be thoroughly screened for GERD to minimize the risk of long-term complications. Furthermore, patients diagnosed with GERD should be screened for depression and anxiety.

## Introduction

Gastroesophageal reflux disease (GERD) is among the most prevalent gastrointestinal (GI) conditions globally, with approximately 10-20% of the western population suffering from it [1,2]. It is a chronic GI disease characterized by regurgitation of gastric contents and acid from the stomach into the esophagus, leading to chest discomfort, dyspepsia, bad breath, and heartburn [3]. GERD is suggested to have multifactorial pathogenesis with decreased pressure at the lower esophageal sphincter (LES), with delayed gastric emptying playing a significant role [3-5]. Other risk factors include hiatal hernia, obesity, obstructive sleep apnea, and helicobacter pylori gastritis. The majority of GERD patients get symptomatic relief with an antacid, but, in some cases, the response to antacid in addition to proton pump inhibitors is low. GERD significantly impacts the quality of life and precipitates poor mental well-being [6,7].

Psychological factors, including stress and anxiety, may contribute to GI disorders. Several studies have found a strong link between psychological disorders and GI functionality. Stress and anxiety may disrupt the normal GI function and lead to several GI disorders, including GERD. Similarly, poor GI health may trigger anxiety and depression in the patients [8,9]. Many studies have been carried out to establish the association between GERD and anxiety and depression. These studies have shown contrasting results. While some report a significant association between GERD and anxiety, others suggest no link between acid reflux and anxiety levels [10-14]. These contrasting results urge the need for further studies to achieve a clearer understanding of the association between GERD and psychological factors. Attributing to the stressful lifestyle, the prevalence of depression and anxiety remains higher in the younger population [15]. Therefore, this study aims to assess the correlation between psychological disorders and GERD in young patients. It shall guide the clinicians toward optimal management of the condition and prevent the psychological factors from worsening the disease symptomatology, treatment outcomes, and its deleterious effect on the quality of life in patients with GERD.

## Materials and methods

This cross-sectional study was conducted in various cities of Pakistan. After taking informed consent, 2,500 participants from the public, in the age group 18 to 40 years, were enrolled in the study. Verbal informed consent was taken from participants. Participants' demographics were noted in a self-structured questionnaire. Diagnosis of GERD was made via the Frequency Scale for the Symptoms of GERD (FSSG) questionnaire [16]. Each response was assigned a score for the frequency of each symptom as follows: 0, never; 1, occasionally; 2, sometimes; 3, often; and 4, always. Patients with scores more than 10 were labeled as having GERD [16]. Based on the FSSG criteria, patients diagnosed with GERD were labeled as the case group, and those negative were included in the reference group. The Hospital Anxiety and Depression Scale (HADS) was used to assess anxiety and depression. Cut-off score was less than 8 on each subscale of anxiety and depression [14]. The interviewer filled the questionnaire after explaining each question to participants.

Statistical analysis was performed using the Statistical Package for Social Sciences® (SPSS) software version 23.0 (IBM Corp., Armonk, NY, USA). Categorical data such as gender, age distribution, anxiety, and depression were presented as frequency and percentages. The chi-square was applied to compare the prevalence of depression and anxiety in participants with and without GERD. A p-value of less than 0.05 meant that the difference between both groups is significant, and the null hypothesis will be rejected.

## Results

GERD was diagnosed in 401 (16.0%) participants. It was more common in males compared to females (62.5% vs. 37.4%; p < 0.01). Smoking was significantly more common in GERD-positive participants compared to GERD-negative participants (49.8% vs. 14.9%; p < 0.01) (Table 1).

**Table 1:** Demographics of the participants BMI, body mass index, GERD, gastroesophageal reflux disease

Characteristics	GERD Positive (n = 401)	GERD Negative (n = 2,099)	p-Value
Gender (%)			
Male	251 (62.5%)	1,001 (47.6%)	<0.01
Female	150 (37.4%)	1,098 (52.4%)	
Age in years (%)			
18-30	271 (67.5%)	974 (46.4%)	<0.01
31-40	130 (32.5%)	1,125 (53.6%)	
Smoking (%)			
Yes	200 (49.8%)	313 (14.9%)	<0.01
No	201 (50.2%)	1,786 (85.1%)	
BMI more than 25 kg/m^2^			
Yes	191 (47.6%)	541 (25.7%)	<0.01
No	210 (52.4%)	1,558 (74.3%)	

Anxiety was significantly more common in participants with GERD (40.3% vs. 19.5%; p < 0.01). Similarly, participants with GERD had a higher prevalence of depression compared to participants without GERD (42.6% vs. 18.3%; p < 0.01) (Table 2).

**Table 2:** Prevalence of anxiety and depression using the HADS score GERD, gastroesophageal reflux disease, HADS, Hospital Anxiety and Depression Scale

HADS Score	Anxiety			Depression		
	GERD Positive (n = 401)	GERD Negative (n = 2,099)	p-Value	GERD Positive (n = 401)	GERD Negative (n = 2,099)	p-Value
Less than 8	162 (40.3%)	411 (19.5%)	<0.01	171 (42.6%)	385 (18.3%)	<0.01
More than 8	139 (59.7%)	1,688 (80.5%)		230 (57.4%)	1,714 (81.7%)	

## Discussion

Our study demonstrated that anxiety and depression were more common in GERD-positive participants in comparison to those without GERD. Moreover, our study indicated that GERD was more prevalent among smokers and male participants.

In compliance with this study, Oh et al. also found an association of depression and anxiety with GERD [14]. Similarly, Yang et al. showed the negative impact of mental disorders on people having GERD symptoms [17]. A high body mass index (BMI) was a common factor of GERD in our study. Obesity causes an elevation in the gastroesophageal pressure, leading to the development of hiatal hernia, which is associated with esophagitis [18]. Moreover, smoking plays a significant role in causing GERD by lowering LES resting pressure, as proved in our study [19]. The anti-reflux mechanism of the esophagus consists of the LES, the angle of His, and muscles of the diaphragm. GERD occurs when gastric acid enters the distal esophagus due to inappropriate relaxation of the LES, further leading to irritation due to the stimulation of chemoreceptors [20]. Some of the risk factors associated with GERD are smoking, high BMI, anxiety, and depression [21]. These patients often face social interaction problems, and consistent reflux can lead to chronic fatigue and sleeping difficulty. This further triggers anxiety and depression symptoms. Hence, identifying triggering factors can help such patients in reducing anxiety, as patients will be proactive in controlling their daily activities [22]. This study will assist people in identifying lifestyle and behavioral modifications to reduce GERD symptoms. These modifications include dietary modification, smoking cessation, and weight reduction. Moreover, it further highlights the fact that people with symptoms should be encouraged for GERD screening and to educate them regarding triggering factors.

This is the first large-scale study on GERD and its association with anxiety and depression in the local setting, targeting the young population. The sample was collected from various cities and thus was diverse. However, since it was a cross-sectional survey, the association between GERD and psychological symptoms could not be established definitely. Further large-scale prospective studies are needed to confirm the association between GERD and psychological diseases.

## Conclusions

There is a positive correlation between GERD and anxiety and depression. Our study found that GERD is highly prevalent in the young population. We speculate that the rigorous lifestyle and high prevalence of depression and anxiety among the young population contribute to symptoms of GERD. Therefore, to minimize the risk of long-term complications, the young population must be screened for GERD. Furthermore, patients diagnosed with GERD should undergo screening for depression and anxiety.
